# Integrating Chronic Obstructive Pulmonary Disease Treatment With 8-Week Tai Chi Chuan Practice: An Exploration of Mind-Body Intervention and Neural Mechanism

**DOI:** 10.3389/fnhum.2022.849481

**Published:** 2022-05-06

**Authors:** Haoran Shen, Li-Zhen Chen, Zhuoer Hu, Xiaoyan Yao, Tao Yang, Lan Zhang, Qiang Tu, Guangxi Li, Gao-Xia Wei

**Affiliations:** ^1^CAS Key Laboratory of Mental Health, Institute of Psychology, Chinese Academy of Sciences, Beijing, China; ^2^Department of Psychology, University of Chinese Academy of Sciences, Beijing, China; ^3^Sino-Danish College, University of Chinese Academy of Sciences, Beijing, China; ^4^Sino-Danish Center for Education and Research, Beijing, China; ^5^Department of Pulmonary Medicine, Guang’anmen Hospital, China Academy of Chinese Medical Sciences, Beijing, China; ^6^Department of Neurology, Jingzhou No. 1 People’s Hospital and First Affiliated Hospital of Yangtze University, Jingzhou, China

**Keywords:** chronic obstructive pulmonary disease, resting fMRI, Tai Chi, frontal lobe, mind-body intervention

## Abstract

**Objective:**

This study aims to explore the effect of integrating routine treatment with Tai Chi Chuan (TCC) intervention on the clinical symptom of patients with Chronic Obstructive Pulmonary Disease (COPD) from clinical and neurological perspectives.

**Methods:**

Twenty patients with COPD were recruited for regular treatment combined with 8-week TCC rehabilitative practice. Clinical symptoms were evaluated by Chronic Obstructive Pulmonary Symptom Assessment Scale (CAT) and Modified Dyspnea Scale (mMRC) at baseline and after treatment. Resting-state MRI scan was also performed with multiline T2-weighted echo-planar imaging (EPI) to acquire their functional images before and after the treatment. TCC rehabilitation involved a total of 8 weeks of practice with 90 min per session, three times a week.

**Results:**

After an 8-week integration routine treatment with TCC practice, the patient’s clinical symptoms improved significantly. Imaging analysis showed that COPD patients exhibited decreased Degree of Centrality (DC) in the right inferior frontal gyrus (IFG), right middle frontal gyrus, bilateral cingulate cortex, bilateral precuneus, and right precentral gyrus. Moreover, correlation analysis found that the decreased DC in the right IFG was positively correlated with the CAT improvements.

**Conclusion:**

The routine treatment involving TCC rehabilitation practice could improve the clinical symptoms of patients with COPD. The right IFG might be a key brain region to contribute to the neural mechanism underlying integrative intervention on the clinical symptoms in COPD. These findings provide neurological evidence for treating COPD rehabilitation practice with mind-body practice based on Chinese culture to some extent, which also advances the understanding of the efficacy of TCC as the adjuvant technology from a neuroscience perspective.

**Clinical Trial Registration:**

[http://www.chictr.org.cn/showproj.aspx?proj=45189], identifier [ChiCTR1900028335].

## Introduction

According to the World Health Organization (WHO), chronic obstructive pulmonary disease (COPD) has become the fourth largest chronic non-communicable disease and is predicted to be the third leading cause of mortality by 2030 (Higginson and Parry, 2018), which seriously threaten human health and survival in the globe. COPD is a chronic inflammatory lung disease that causes airflow obstruction. It is accompanied by a variety of clinical symptoms including coughing, shortness of breath, and chest tightness, which might lead to poor quality of life, impaired social function, and death (Watz et al., 2008).

Brain imaging evidence has shown that patients with COPD exhibit significant atrophy in the frontal lobe, cingulate cortex, anterior insular lobe, and hippocampus, along with aberrant brain function, with decreased cortical volume and density in the local brain structures (Zhang H. et al., 2013; Cleutjens et al., 2017). A recent study using fMRI observed that the functional connectivity between the right superior temporal gyrus, right inferior frontal gyrus (IFG), right paracentric lobule, and the right cingulate gyrus in patients with COPD were lower than those in healthy subjects (Li et al., 2020). Another PET study also found a similar abnormality, showing that the cerebral perfusion of the frontal lobe and parietal lobe were significantly lower in patients with COPD relative to healthy individuals (Ortapamuk and Naldoken, 2006). These studies suggest extensive abnormalities of anatomical structure and functional organization in patients with COPD.

Tai Chi Chuan (TCC), a typical mind-body integrative practice based on Chinese traditional culture, is regarded as an alternative and complementary medication. It emphasizes the “unity of body and mind.” Some studies found that TCC played an important role in “cultivating the mind” (Dechamps et al., 2009). This form of aerobic exercise requires the brain to be in a highly focused and relaxed state, coupled with rhythmic breathing regulation. Since it involves multiple components, including mindfulness, aerobic exercise, and breathing training, it could reshape the brain structure and alter brain function associated with optimized cognitive function and emotional health (Wei et al., 2016; Zhang et al., 2019; Yao et al., 2021). Previous studies demonstrated the role of physical exercise in improving clinical symptoms in patients with COPD (Emery et al., 1998; Ngai et al., 2016). However, the clinical efficacy of integrating routine treatment of COPD with TCC practice remains largely unknown. The neural mechanism underpinning its effects is also not clear.

With the development of resting-fMRI, degree of centrality (DC) has recently gained great attention in clinical studies. This graph-based measurement of network organization reflects the number of instantaneous functional connections between a region and the rest of the brain within the entire connectivity matrix of the brain (Guo et al., 2016). It has been widely used in neurological disorders, such as COPD, Parkinson’s disease, Alzheimer’s disease, and schizophrenia (Wang et al., 2019; Li et al., 2020; Rong et al., 2021). The DC approach was applied here to investigate altered brain functions after intervention in patients with COPD who received TCC rehabilitation practice. Moreover, we examined if the altered brain function was associated with the improved clinical symptom.

## Methods

### Patients

Twenty patients with COPD were recruited from the Department of Respiratory Medicine, Guang’anmen Hospital, China Academy of Chinese Medical Sciences from January 2019 to December 2019. The final sample included seventeen patients since three of them dropped out in the follow-up assessment.

The Global Initiative for COPD in 2019 (GOLD 2019) guidelines were used as clinical diagnosis of COPD. Mild to moderate COPD. The inclusion criteria were as follows: (1) patients aged from 55 to 80 years; (2) patients who had no regular exercise in the past 6 months. The exclusion criteria are as follows: (1) patients with history of hearing or vision problems, physical injury, seizures, metal implants, head trauma with loss of consciousness, or pregnancy; (2) patients with severe pulmonary diseases with somatic comorbidities such as cardiovascular, liver, kidney, and hematopoietic diseases; (3) patients with severe bone and joint diseases or osteoporosis; (4) patients with neurological and psychotic disorders, such as Alzheimer’s disease, psychosis, and emotional disorders, etc.; and (5) patients who cannot follow the doctor’s advice or examination as planned.

### Clinical Assessments

All patients underwent systematic evaluation of clinical symptoms before and after the 8-week TCC practice. The COPD Assessment Test (CAT) was used to evaluate the chronic obstructive pulmonary symptom, which included eight questions graded from 0 to 5 for cough, phlegm, chest tightness, exertional dyspnea, limited activity, confidence in leaving home, sleeplessness, and loss of energy (Jones et al., 2009). The modified Medical Research Council (mMRC) scale was used to measure dyspnea on exertion, with a higher score indicating worse dyspnea (Paternostro-Sluga et al., 2008).

### Tai Chi Chuan Rehabilitation Practice

**FIGURE 1:**
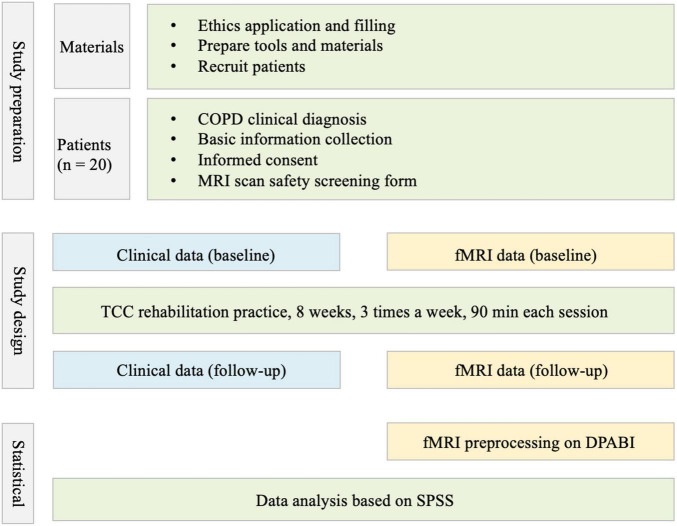
The routine of study.

All patients received routine treatment combined with TCC practice for 8 weeks. In the routine treatment, inhalation preparation was used regularly according to the GOLD 2019 guidelines. TCC practice intervention lasted for 8 weeks, three times a week. Each session included a 20-min warm-up exercise, 10-min whole-body exercise, 55 min of 24-style TCC, and 5 min of cool down. All patients were divided into three groups supervised by skilled TCC instructors (Figure 1).

### Resting-State fMRI Data Acquisition and Pre-processing

All the MRI data were acquired on a GE 3 Tesla MRI scanner (MAGNETOM Trio TimSystem) before and after TCC practice. During brain scanning, patients were instructed to keep their eyes closed, move as little as possible, and try not to engage in any mental activity. Sponge earplugs and headphones were provided for patients to use to reduce noise during scanning. High-resolution structural images were acquired using a magnetization-prepared rapid gradient echo (MPRAGE) three-dimensional T1-weighted sequence [Repetition time (TR)/echo time (TE) = 2,530/3.45 ms, flip angle (FA) = 7.0°, field of view (FOV) = 220 mm × 220 mm, matrix = 256 × 256, slice thickness = 1 mm]. Resting-state functional images were obtained using an Echo Planar Imaging (EPI) with the following scan parameters: Repetition time (TR)/echo time (TE) = 2,000/30 ms, flip angle (FA) = 9.0°, field of view (FOV) = 220 mm × 220 mm, matrix = 64 × 64, slice thickness = 3 mm. This sequence contained 300-time points, and the total scanning time was about 10 min.

Data Processing and Analysis for Brain Imaging (DPABI) based on the MATLAB platform were used to pre-process fMRI data. The following steps were performed sequentially: (1) DICOM raw data was converted to NIFTI format; (2) the first 10 time points were removed to allow the signal to reach equilibrium; (3) slice timing correction was performed; (4) realignment for head motion correction was done, excluding patients whose head motion exceeded 2.0 mm or 2.0 degrees of axial rotation; (5) the regression covariates, including cerebral white matter signal, cerebrospinal fluid signal, and head motion parameters.; (6) spatial normalization: functional images were normalized to the standard Montreal Neurological Institute (MNI) template using DARTEL, and were resampled to 3 mm × 3 mm × 3 mm; (7) de-linearization drift and filtering (0.01-0.1 Hz); and (8) based on pre-processing, DC values reflecting the connectivity properties of the functional brain network were calculated using Data Processing Assistant for Resting-State fMRI (DPARSF)^1^ according to previous studies (Zuo et al., 2011; Li et al., 2016).

### Statistical Analysis

The statistical analysis was performed on Statistical Package for the Social Sciences (SPSS) 26.0 (IBM Corp., Armonk, NY, United States). Regarding clinical characteristics changes, the CAT and mMRC scores were in line with normal distribution. Thus, a paired-samples *t*-test was conducted to analyze symptom improvements before and after TCC rehabilitation practice. As for alterations in brain functions, Gaussian random field (GRF) correction (voxel *p* < 0.05, cluster *p* < 0.05) was performed on DC values after the paired-samples *t*-test, and the brain regions with significant alterations were extracted. Moreover, Spearman rank correlation was used to analyze correlations between DC value alterations and clinical improvements. Statistical significance was defined as *p* < 0.05.

## Results

### Clinical Symptoms Improvements After Intervention

The primary outcome was the improvement of clinical symptoms in COPD. Paired-samples *t*-test showed that after 8-week intervention, CAT score significantly improved after treatment compared to baseline [t_(16)_ = −5.17, *p* < 0.001], but there was no significant change in mMRC [t_(16)_ = −1.39, *p* > 0.05] (Table 1).

**TABLE 1:** Clinical symptoms before and after integrating routine treatment with Tai Chi Chuan (TCC) practice.

	Before (*n* = 20)	After (*n* = 17)	t	*p*
Age	66.90 ± 7.17	65.82 ± 7.02		
Sex (M/F)	15/5	13/4		
CAT	19.75 ± 4.31	16.50 ± 3.06	−5.17	<0.001
mMRC	1.42 ± 0.67	1.17 ± 0.58	−1.39	0.19

*CAT, chronic obstructive pulmonary symptom assessment scale; mMRC, modified dyspnea scale.*

### Alterations in Degree of Centrality After Intervention

**FIGURE 2:**
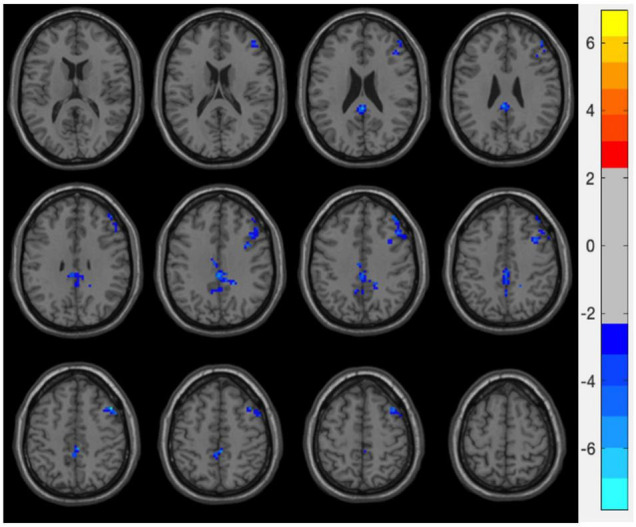
Degree centrality (DC) vales alterations after integrating routine treatment with Tai Chi Chuan (TCC) practice. After practice, DC values in the right inferior frontal gyrus (IFG), right middle frontal gyrus, bilateral cingulate cortex, bilateral precuneus, and right precentral gyrus were decreased.

After GRF correction, the DC value significantly decreased in the right IFG, right middle frontal gyrus, bilateral cingulate cortex, bilateral precuneus, and right precentral gyrus (*p* < 0.05) after intervention (Figure 2 and Table 2).

**TABLE 2:** Brain area of degree centrality (DC) changes after integrating routine treatment with Tai Chi Chuan (TCC) practice.

	Brain area	Peak MNI			Peak value
		
		X	Y	Z	
DC	Inferior frontal gyrus	51	27	30	−2.31
	Cingulate cortex	18	−45	33	−2.31

*DC, degree centrality.*

### Relationships Between DC Alterations and Clinical Response

**FIGURE 3:**
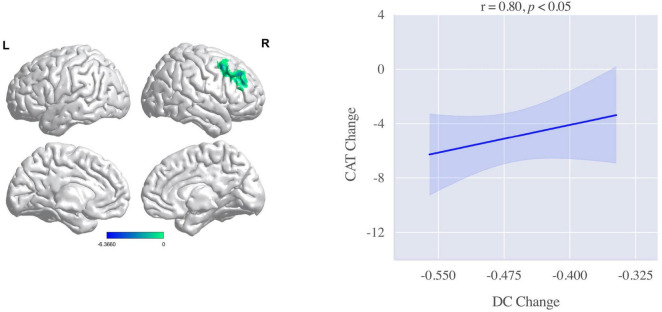
Correlation between degree centrality (DC) changes of the right inferior frontal gyrus (IFG) and chronic obstructive pulmonary disease (COPD) assessment test (CAT) improvements. The DC changes in the right IFG was positively associated with the improvements in CAT after intervention.

To explore the neural mechanism for clinical symptoms improvements, we calculated the correlations between DC changes and clinical symptoms improvements. The brain regions significantly decreased in DC after intervention were used as regions of interest (ROI). The decreased values were extracted from all patients. As shown in Figure 3, the correlational analysis indicated that decreased DC in the IFG was positively associated with CAT improvements (*r* = 0.80, *p* < 0.05).

## Discussion

To our knowledge, this was the first study to investigate the effect of TCC practice on brain function associated with clinical symptoms improvements in COPD. We found that routine treatment integrating with TCC rehabilitation practice could significantly improve pulmonary symptoms measured by CAT in COPD. Moreover, DC values were decreased in the right IFG, right middle frontal gyrus, bilateral cingulate cortex, bilateral precuneus, and right precentral gyrus after the treatment. Intriguingly, the improved pulmonary symptoms were positively associated with the decreased DC value in the IFG, which suggested that IFG might be the key brain region related to the neural mechanism of the effects of routine treatment integrating with TCC practice on COPD. These results demonstrated the therapeutic effect of traditional body-mind regulation techniques on COPD. Most importantly, these findings are of great implications for the treatment of COPD from a Chinese culture-based approach.

In this study, the most intriguing finding was that the decreased DC value of the right IFG after the intervention was associated with chronic obstructive pulmonary symptoms measured by CAT, which suggested that the neural mechanism of routine treatment integrated with TCC practice on COPD decrease DC values in the IFG. Mounting evidence has demonstrated that IFG plays an essential role in pulmonary function and respiration. In healthy adults, the respiration volume was negatively associated with the blood-oxygen-level-dependent (BOLD) signal in the IFG, superior/middle temporal gyrus, inferior parietal lobule, and visual cortex (Yuan et al., 2013). For immigrants who are adapted to high altitude, their impairments in vital capacity were correlated with increased volumes in the right middle frontal gyrus and right parahippocampal gyrus (Zhang J. et al., 2013). Due to the important role of IFG in pulmonary function, the impairments of pulmonary function in COPD may be closely associated with the abnormalities in the IFG anatomy and function, which is consistent with previous studies. A resting-state fMRI found that compared with healthy controls, functional connectivities between the supplementary motor area and the right IFG, anterior cingulate gyrus, right insula, and limbic lobe were decreased in patients with COPD (Li et al., 2019). Another MRI study used voxel-based morphometry and observed that the gray matter density of the right IFG was reduced in patients with COPD. In addition, patients with severe symptoms of COPD exhibited more extensive reductions than those with moderate clinical symptoms (Yin et al., 2019).

Previous studies demonstrated that TCC has a positive effect on alleviating pulmonary symptoms and improving the respiratory function of patients with COPD (Leung et al., 2013; Ng et al., 2014). In addition, in asthmatic children, after 12-week TCC training, the fractional exhaled nitric oxide (FeNO) level was decreased, and peak expiratory flow rate (PEFR) and forced expiratory volume in 1 s (FEV1)/forced vital capacity (FVC) was increased, suggesting that the pulmonary function of asthmatic children significantly improved (Lin et al., 2017). During TCC practice, a good coordinated respiratory system is a requisite for performing whole movements that need complex motor skills. As such, individuals with a higher level of TCC practice exhibit greater breath amplitude and better cardiopulmonary function (Wei et al., 2016). Furthermore, some brain imaging evidence have demonstrated that TCC practice could reshape the brain structure and optimize regional brain function, mainly the frontal lobe (Yao et al., 2021). For instance, 20-week TCC practice decreased resting-state functional connectivity between the dorsolateral prefrontal cortex, left superior frontal gyrus, and the anterior cingulate cortex in older adults (Tao et al., 2017). A cross-sectional study revealed that compared with controls, TCC practitioners had lower middle frontal gyrus voxel-mirrored homotopic connectivity (VMHC) (Chen et al., 2020). In this study, we found the decreased DC values in the right IFG after TCC practice was positively associated with pulmonary symptom improvements, which indicated that the decreased IFG DC values may portray the neural mechanism of the improvements of the pulmonary symptoms in COPD.

Although this study has potential significance, its main limitations should be acknowledged. First, the sample size was relatively limited. A larger sample size of replication studies is needed in future studies. Second, this study lacked a control group with routine treatment. The alterations in the clinical symptoms and DC values may be caused by both the routine treatment and TCC practice. Thus, caution should be taken when concluding the neural mechanism of TCC practice in patients with COPD. Future studies should use randomized controlled trials to further explore the effect of mind-body practice in the treatment of COPD.

## Conclusion

This study found that an 8-week integration routine treatment with TCC practice significantly improved lung function in patients with COPD. The right IFG might be a key brain region to contribute to the neural mechanism underlying the integration of routine treatment with TCC intervention on clinical symptoms of COPD. These findings provide neurological evidence for treating COPD rehabilitation practice with mind-body practice based on Chinese culture to some extent, which also advances the understanding of the efficacy of TCC as the adjuvant technology from a neuroscience perspective.

## Data Availability Statement

The original contributions presented in the study are included in the article/supplementary material, further inquiries can be directed to the corresponding author/s.

## Ethics Statement

The studies involving human participants were reviewed and approved by the Guang’anmen Hospital, China Academy of Chinese Medical Sciences. The patients/participants provided their written informed consent to participate in this study. Written informed consent was obtained from the individual(s) for the publication of any potentially identifiable images or data included in this article.

## Author Contributions

G-XW and GL designed the study, reviewed, and revised the manuscript. TY, LZ, QT, L-ZC, and XY were responsible for recruiting the patients, performing the clinical rating, and collecting the clinical data. HS, L-ZC, and ZH cleaned data and did the statistical analysis. HS and G-XW wrote the manuscript. All authors contributed to the article and approved the submitted version.

## Conflict of Interest

The authors declare that the research was conducted in the absence of any commercial or financial relationships that could be construed as a potential conflict of interest.

## Publisher’s Note

All claims expressed in this article are solely those of the authors and do not necessarily represent those of their affiliated organizations, or those of the publisher, the editors and the reviewers. Any product that may be evaluated in this article, or claim that may be made by its manufacturer, is not guaranteed or endorsed by the publisher.

## Footnotes

1
HTTP://rfmri.org/DPARSF
